# Functionalized polystyrene nanoparticles as a platform for studying bio–nano interactions

**DOI:** 10.3762/bjnano.5.250

**Published:** 2014-12-15

**Authors:** Cornelia Loos, Tatiana Syrovets, Anna Musyanovych, Volker Mailänder, Katharina Landfester, G Ulrich Nienhaus, Thomas Simmet

**Affiliations:** 1 Institute of Pharmacology of Natural Products & Clinical Pharmacology, Ulm University, Helmholtzstr. 20, D-89081 Ulm, Germany; 2 Max-Planck-Institute for Polymer Research, Ackermannweg 10, D-55128 Mainz, Germany; 3 Institute of Applied Physics, Karlsruhe Institute of Technology (KIT), Wolfgang Gaede-Str. 1, D-76131 Karlsruhe, Germany,; 4 Department of Physics, University of Illinois at Urbana-Champaign, 1110 West Green Str. Urbana, Illinois 61801, United States

**Keywords:** amino groups, apoptosis, carboxyl groups, cell proliferation, leukemia cell lines, macrophages, mTOR, polystyrene nanoparticles

## Abstract

Nanoparticles of various shapes, sizes, and materials carrying different surface modifications have numerous technological and biomedical applications. Yet, the mechanisms by which nanoparticles interact with biological structures as well as their biological impact and hazards remain poorly investigated. Due to their large surface to volume ratio, nanoparticles usually exhibit properties that differ from those of bulk materials. Particularly, the surface chemistry of the nanoparticles is crucial for their durability and solubility in biological media as well as for their biocompatibility and biodistribution. Polystyrene does not degrade in the cellular environment and exhibits no short-term cytotoxicity. Because polystyrene nanoparticles can be easily synthesized in a wide range of sizes with distinct surface functionalizations, they are perfectly suited as model particles to study the effects of the particle surface characteristics on various biological parameters. Therefore, we have exploited polystyrene nanoparticles as a convenient platform to study bio–nano interactions. This review summarizes studies on positively and negatively charged polystyrene nanoparticles and compares them with clinically used superparamagnetic iron oxide nanoparticles.

## Review

### Applications of polystyrene

**Figure 1:**
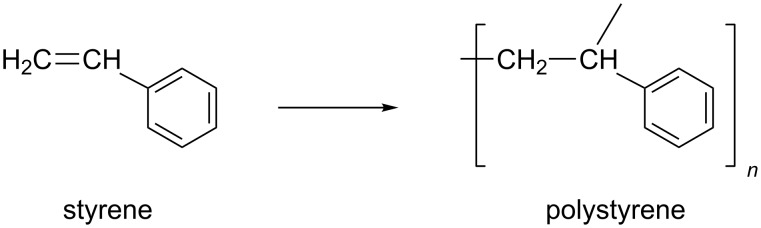
Polystyrene synthesis.

Polystyrene, one of the most extensively used types of plastic [1], is an aromatic polymer obtained by polymerization of styrene monomers (Figure 1). For the industrial mass production of polystyrene, styrene monomers are produced by the catalytic dehydrogenation of ethylbenzene which, in turn, is synthesized by the petrochemical industry [1].

Commonly used polystyrene is being molded or expanded to foams. Such a thermoplastic polymer has an atactic conformation without crystalline regions. Hence, the homopolymer is transparent, durable, and can be colored very easily. Polystyrene is hardly biodegradable facilitating its use in the food and medical product and devices industry. Rigid and tough, closed-cell foam, called pre-expanded, polystyrene is used for disposable trays, plates, bowls and cups, for food storage and transport, but also for containers of non-food articles such as cosmetics, pharmaceuticals and cleaning agents. Toys, paper clips, pegs, and office supplies are also often made from polystyrene [1–3]. Due to the biocompatibility of polystyrene, it is widely used for laboratory equipment. After production, equipment made out of polystyrene can be easily sterilized by UV light or ethylene oxide and surface-modified to introduce various polar groups to suit laboratory needs [4].

The number of polystyrene applications has steadily grown over the past 20 years. Although polystyrene can be recycled, only a small portion of the produced polystyrene is actually recycled. According to the U.S. Environmental Protection Agency’s Municipal Solid Waste statistics of 2005, solid non-recycled polystyrene waste amounted to almost 2.6 million tons alone in the USA [5]. Even though it has been reported that the Actinobacteria strain *Rhodococcus ruber* may degrade a thin film of polystyrene, the rate of polystyrene degradation with 0.04 to 0.57% during several weeks in soil are extremely low rendering polystyrene basically non-biodegradable [6–7]. Non-recycled polystyrene disposables pose a great problem due to the long-lasting environmental pollution [5].

### Polystyrene safety

The toxicity of polystyrene, as a material in so many objects of daily use, is controlled by various agencies and authorities. The main source of polystyrene toxicity is its monomer styrene, which may be released during polystyrene heating or manufacture [8]. The Reference Concentration (RfC) by the Environmental Protection Agency (EPA) for chronic toxicity value of styrene based on the studies by Mutti et al. is 300 ppm (1,000 µg/m^3^) [9]. If the level of styrene in the air exceeds this value, there is a possibility of adverse health effects. Among others, the EPA has declared styrene as a suspected carcinogen and a suspected toxin to the gastrointestinal tract, kidney, and respiratory system [10–11]. Although there is no clear proof for styrene being carcinogenic [12], the International Agency for Research on Cancer IARC has categorized styrene to class 2b meaning possibly carcinogenic to humans. One of the most comprehensive reviews by Brown et al. [13] questions putative developmental and fertility effects of styrene as well. In addition, air levels of styrene in the polymer industry usually do not exceed 20 ppm [12], which are much lower than those, which may cause any health hazard.

Polystyrene is thermally relatively stable. Although, almost no degradation of pure polystyrene occurs at temperatures below 200 °C, trace amounts of styrene, ethylbenzene, and cumene could be detected at this temperature by analytical methods. After exposure for 2 h at temperatures above 330 °C, styrene fully decomposes producing mainly styrene monomer [8]. When compared with other common natural and synthetic building materials, the thermal decomposition products of polystyrene appear to be among the least toxic [8].

The estimated amount of residual materials including styrene, which are able to diffuse from the polystyrene packaging, demonstrates a relatively high safety profile for polystyrene. EDI (estimated intake) of styrene from polystyrene is 9 µg/person/day [14], the acceptable daily intake (ADI) reported by the FDA is 90,000 µg/person/day [15]. Accordingly, the use of polystyrene for packaging material presents no cause for health or safety concerns. Additionally, a review by the experts from the Harvard Center for Risk Assessment concluded that there is no reason for concern from exposure to polystyrene materials used in food-contact applications [16].

Due to its inertness and biocompatibility, polystyrene together with polycarbonate is widely used for the production of biomedical devices and laboratory equipment. The polystyrene surface, which is hydrophobic in nature, can be easily modified, for example, by oxidation creating surfaces highly suitable for the efficient growth of cells in culture [17]. Such charged surfaces could be sterilized through UV light and ethylene oxide with no adverse effects on cell growth [4].

### Nanoparticles

Nanoparticles, limited in size to 100 nm in either two or three dimensions [18], fill the gap between molecules and bulk material and between biomolecules and cells. The atoms located at the surface of a nanoparticle have less neighbors than atoms of a bulk material, resulting in lower binding energy per atom with decreasing particle size. A consequence of the reduced binding energy per atom is a reduction of the melting point temperature with the particle radius [19]. Nanoparticles have a very large surface area compared to their volume, which can interact with biological systems, and thereby offers unique application possibilities [20]. All these factors affect the chemical reactivity of nanosized materials as well as their mechanical, optical, electric, and magnetic properties [21].

Nanoparticles offer numerous possibilities of application as catalysts for industrial usage, fuel additives for catalysis, additives in sunscreens for UV protection, or in the textile industry. One of the most promising fields of nanotechnology is drug delivery and drug targeting. Hydrophobic drugs are poorly soluble in biological media, other drugs lack gastric acid resistance and cannot be applied orally. Such drugs could be encapsulated within nanoparticles protecting the drug, generating a new hydrophilic surface, improving pharmacokinetics and targeting the drug to distinct cells and tissues This would enable a reduction of the drug dosage thereby improving the safety profile by decreased undesirable side effects, because the latter are dose-dependent in about 95% [22].

#### Nanoparticle safety

The special physicochemical properties of nanoparticles gave rise to concerns about health effects, which cannot be predicted just by adopting the safety risks of the corresponding bulk material. Pioneering studies on the toxic effects of ultrafine airborne particles (nanoparticles) were conducted already more than 20 years ago [23]. Since then, it has been demonstrated that intratracheally injected airborne nanoparticles such as TiO_2_, carbon black and mineral dust could induce lung injury. Changes in material, size or the surface of the particles results in alternation of the toxicity, which makes it unlikely to integrate nanoparticle toxicology in a single unifying concept [24].

Macrophages are phagocytes that are equipped with specific receptors, which enable the recognition and internalization of particulate matter including nanoparticles. As a consequence, macrophages accumulate with time a main portion of nanoparticles incorporated by the body [25]. Thus, the clinically approved superparamagnetic iron oxide (SPIO) MRI contrast agent Resovist^TM^ is taken up after intravenous injection mostly by liver and spleen macrophages and is retained there for more than two weeks [26]. In contrast to other cells, macrophages express scavenger receptor A on their surface, which mediates endocytosis of diverse ligands including modified low density lipoproteins and which has been implicated in the development of atherosclerosis [27]. In vitro studies showed that this receptor is engaged in the internalization of negatively charged Resovist^TM^, a SPIO of 20–60 nm in size, by human macrophages via clathrin-mediated endocytosis. Hence, the uptake of negatively charged nanoparticles of this size occurs in a specific, receptor-mediated manner, which is characterized by the polymerization of clathrin units, membrane deformation, and intracellular signaling, which could all be integrated into a mathematical model describing these processes [28]. Of note, depending on the nature of the nanoparticles and specifically with respect to polymer-coated SPIO, it may be important to test for longer term toxicity beyond the usual 24 or 48 hour time intervals. Thus, long-term incubation with carboxydextran-coated SPIO nanoparticles induced delayed apoptosis in macrophages through the induction of reactive oxygen species (ROS) and the subsequent activation of c-Jun N-terminal kinases (JNK) signaling [29]. A carboxydextran shell around clinically used SPIO delays its cytotoxicity. However, nanoparticles accumulate within lysosomes, in which the lysosomal α-glucosidase degrades the carboxydextran polymer over time liberating finally molecular iron that subsequently catalyzes the generation of ROS in Fenton and Haber–Weiss reactions. Therefore, nanoparticles with thinner shells exhibit a higher cytotoxicity [30]. In line with these molecular mechanisms, ROS scavengers prevented the ROS-based cytotoxicity of the SPIO nanoparticles [29–30].

These in vitro data are highly relevant for in vivo studies, because after intravenous injection, the carboxydextran-coated SPIO accumulate primarily in liver macrophages, so-called Kupffer cells, which constitute only about 2% of all liver cells, and remain there for prolonged periods of time [30]. The SPIO-loaded Kupffer cells undergo apoptotic cell death, which leads to partial depletion of Kupffer cells in the liver of mice five days after injection. The iron-mediated Kupffer cell toxicity in vivo could be prevented by the ROS scavenger edaravone registered in Japan for the treatment of stroke patients confirming that the adverse effects of SPIO contrast agents can be antagonized by pharmacological means [29–30].

This example shows that application of nanotechnology in biomedicine requires precise analysis of interactions between nanoparticles and living cells. The biological effects of nanoparticles depend not only on the particle material and their size, but to a great extent also on the surface chemistry of the particles. Surface functionalization of nanoparticles is crucial for their pharmacokinetics, biocompatibility, and tissue and cell affinity, and may give us valuable clues for the rational design of nanosized medical devices.

### Biological effects of polystyrene nanoparticles

Polystyrene nanoparticles have been used for various applications, such as biosensors [31], in photonics [32], and in self-assembling nanostructures [33]. Polystyrene is biocompatible and is not expected to adversely affect interactions of nanoparticles with biological systems. Specifically surface-modified polystyrene nanoparticles are homogeneous, exhibit a low polydispersity index, and form stable colloids in biological fluids [34]. We have used polystyrene nanoparticles as model particles for our experiments aimed to analyze the effect of different surface functionalization on various biomedical parameters.

Macrophages as key players of innate and adaptive immunity phagocytize cellular debris and pathogens [35]. Hence, they are settling in particularly large numbers in tissues exposed to pathogens; for example, as alveolar macrophages in lungs, as Kupffer cells in the liver, and as sinusoidal lining cells in the spleen. Damaged or infected tissues contain a large number of macrophages, which originate from infiltrated monocytes. Thus, it is most likely that intentionally applied or unintentionally inhaled nanoparticles get in close contact with macrophages in one way or another.

Phagocytosis of debris by phagocytes including macrophages leads to the formation of phagosomes, which subsequently fuse with lysosomes to phagolysosomes. Within the latter, lysosomal enzymes, low pH and the elevated ROS production enable degradation of its contents. According to their functional task, macrophages have a higher potential for ROS generation than other non-phagocytic cells [20]. These specific features of phagocytes clearly underline the importance of toxicological studies using macrophages.

Tumor cell lines are often used as models to study nanoparticle–cell interactions. Many studies analyzing the toxicity of nanoparticles on macrophages have actually been carried out with leukemia cancer cell lines of murine or human origin at different stages of differentiation [36–37] used as macrophage surrogates.

**Figure 2:**
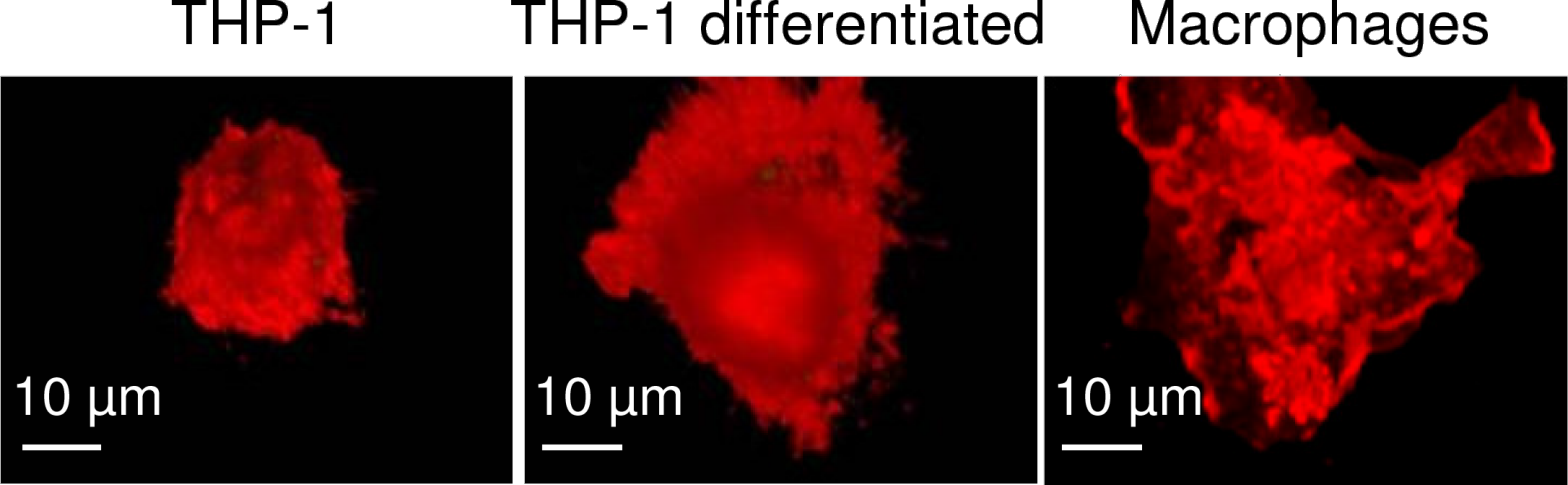
Spinning disc confocal microscopy of acute monocytic leukemia THP-1 cells, differentiated THP-1 cells, and human macrophages. Cell membranes are stained with CellMask (red). Adapted with permission from [43]. Copyright 2011 American Chemical Society.

THP-1 is a cell line from the blood of a boy who suffered from acute monocytic leukemia. Childhood myelomonocytic leukemia is an aggressive clonal disease of pluripotent stem cells, which is clinically characterized by overproduction of monocytic cells that can infiltrate inner organs, such as spleen, liver, and lungs [38]. To mimic the differentiated state of macrophages for in vitro studies, monocytic cells can be further differentiated with phorbol-12-myristate-13-acetate (PMA) or 1,25-dihydroxyvitamin D_3_ [39–40]. Figure 2 shows that the phenotype of human macrophages differs already in terms of morphology and size from that of THP-1 or PMA-differentiated THP-1 cells. THP-1 cells are much smaller and grow in suspension, whereas macrophages and PMA-differentiated THP-1 reside as adherent cells.

In our studies, we used polystyrene nanoparticles of about 110 nm diameter, which were non-functionalized or surface-functionalized either with carboxyl (PS-COOH) or amino (PS-NH_2_) groups, and which carried roughly the same functional group density of 6,000 groups per particle [41–43]. This allowed us to analyze the effect of nanoparticle surface functionalization on cell functions. Particles of about 100 nm have previously been shown to be internalized by cells much more efficient than microparticles, which are taken up primarily by phagocytosis, and also more efficient than particles with a size below 100 nm. It was reported that the uptake of 100 nm particles was 2.3-fold greater than that of 50 nm particles [44].

Both, macrophages and THP-1 cells rapidly internalized 110 nm PS-COOH and PS-NH_2_ nanoparticles. However, the amount of internalized nanoparticles, the uptake kinetics, and its mechanism differed between primary cells and the related THP-1 leukemia cells, whether differentiated or not [43]. Interestingly, also the surface functionalization affected the rate and amount of nanoparticle uptake. Because the uptake mechanism used by the cells was also different for buffer and serum-containing medium, it was obvious that opsonization played a crucial role in the uptake mechanism. In fact, PS-COOH bound more and different proteins in comparison to PS-NH_2_, which is in line with a previous report [45]. The complex dynamics and kinetics of PS protein coronas has only recently been addressed in detail [46]. In any case, macrophages internalized about four times more negatively charged nanoparticles in cell culture medium. By contrast, monocytic leukemia cells, internalized more rapidly positively charged nanoparticles independently of the assay media. The ability of macrophages to preferentially internalize negatively charged nanoparticles over positively charged ones may be unique. Thus, non-phagocytic cells, such as fibroblasts and endothelial cells, took up significantly more positively charged Au NPs than negatively charged Au NPs [47]. This emphasizes that the uptake of nanoparticles is highly cell type-dependent and the expression of specific receptors engaged in the uptake might dictate the rate of particle internalization by a cell. We have shown that in the presence of serum, macrophages take up nanoparticles by phagocytosis using specific interaction with an antibody receptor CD64, which is specifically expressed on the surface of immune cells. In contrast, monocytic leukemia cells internalize nanoparticles by endocytosis. Also in vivo, intravenously injected negatively charged particles accumulate mainly in the liver, known to harbour macrophages of the reticuloendothelial system named Kupffer cells. In contrast, positively charged particles were found mostly in leukemia xenografts [43].

With particles left in the cell culture media, the cellular uptake reaches equilibrium within 24 h. When particles were removed from the media, there was virtually an exponential decrease of the amount of particles in proliferating THP-1 cells. The cells distribute particles between the daughter cells and, thus, decrease the individual nanoparticle load. Thus, after three days, a THP-1 cell holds a third of the amount of particles measured after the first day and after six days there are only about 10% of PS-COOH and about 19% of PS-NH_2_ particles left. Neither THP-1, nor macrophages release nanoparticles back into the culture media, as measured during the six days of cell culture. This indicates that in macrophages, which do not proliferate, the amount of internalized non-biodegradable nanoparticles is not reduced with time.

**Figure 3:**
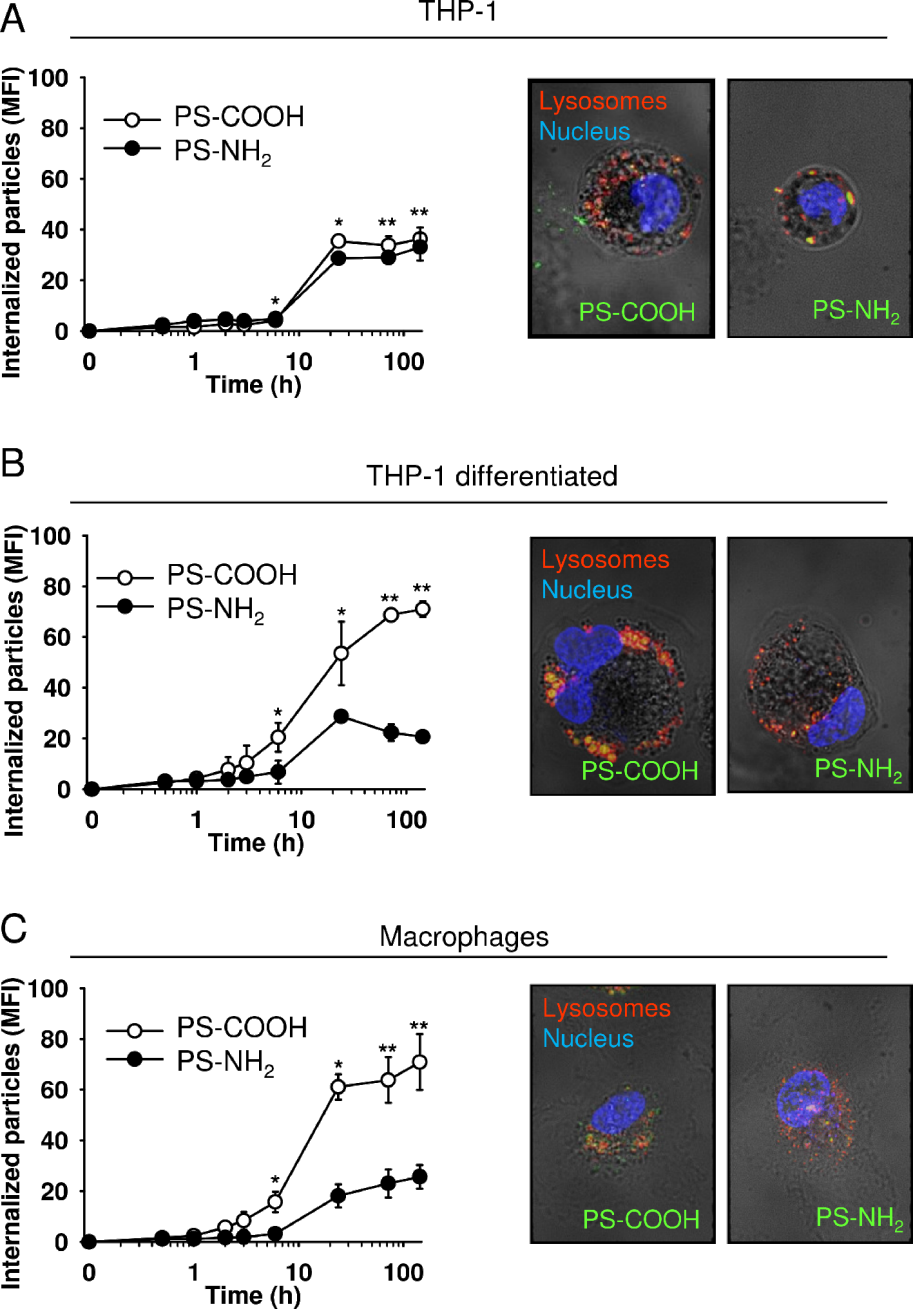
Uptake kinetics and subcellular localization of carboxyl- and amino-functionalized polystyrene nanoparticles by THP-1 (A), differentiated THP-1 cells (B), and human macrophages (C). The cells were incubated with PS-COOH or PS-NH_2_ nanoparticles (each 100 µg/mL) and analyzed by flow cytometry or confocal microscopy (24 h). Results are given as mean ± SEM, *n* = 3, *p < 0.05, **p < 0.01. Nuclei are labeled with HCS NuclearMask™ (blue), nanoparticles are stained with PMI (green), lysosomes are labeled with LysoTracker® Red DND-99 (red). Original magnification 900×. Photomicrographs show overlay with phase contrast images. Adapted with permission from [41]. Copyright 2014 Elsevier.

To investigate the fate of internalized particles, the subcellular compartments were stained with corresponding fluorescent probes. Even though, the particles are taken up by THP-1, differentiated THP-1 cells, and macrophages by different mechanisms, after 24–72 h most of the particles are co-localized with lysosomes independent of the cell type (Figure 3, and [41–43]).

**Figure 4:**
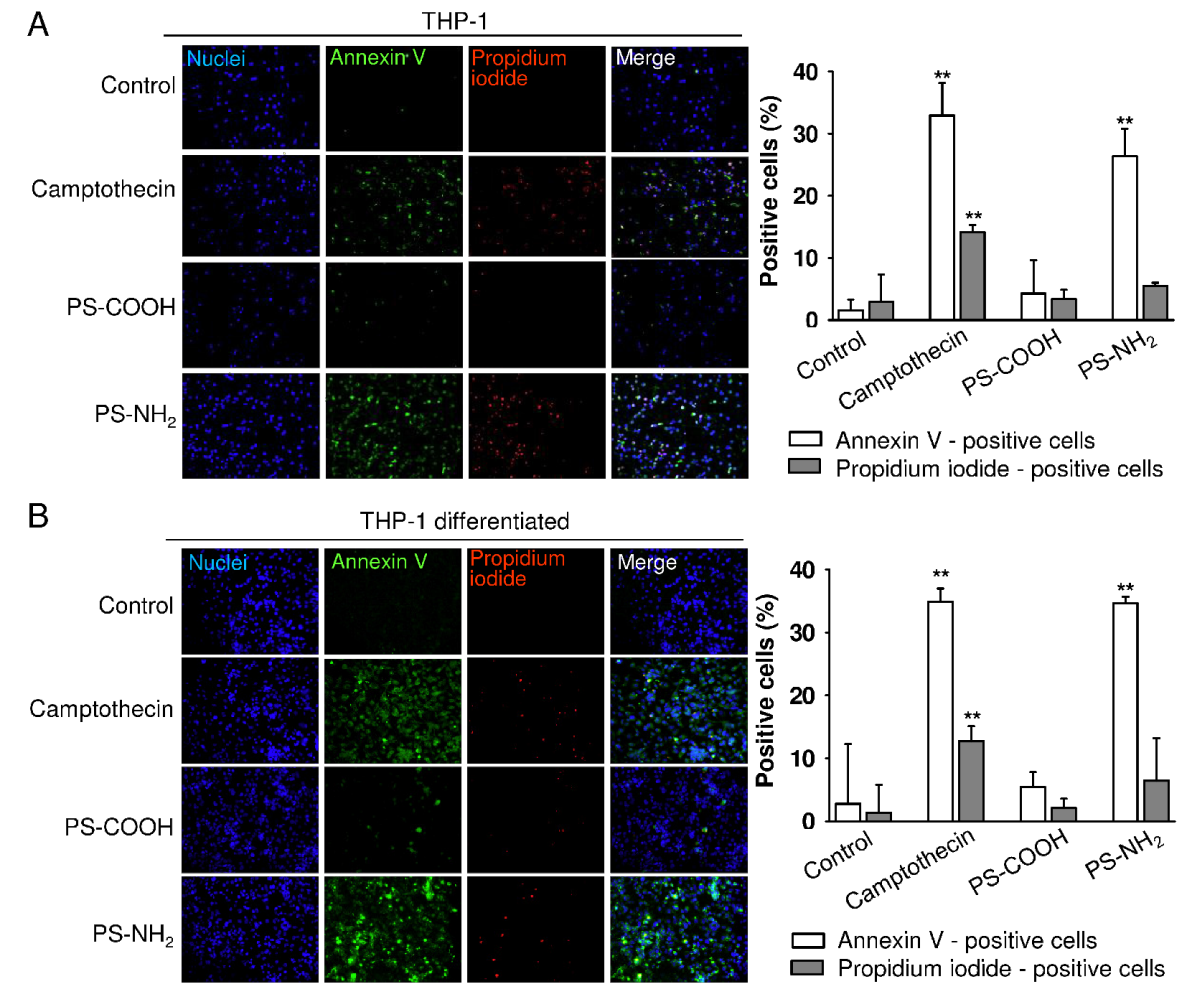
Amino-functionalized polystyrene nanoparticles induce apoptotic cell death. THP-1 (A) and differentiated THP-1 (B) were stimulated with either PS-COOH or PS-NH_2_ (each at 100 µg/mL) for 72 h, analyzed by using fluorescence microscopy and quantified by using ImageJ. The graphs show the amounts of apoptotic (annexin V^+^) and late apoptotic or necrotic (propidium iodide^+^) cells. Camptothecin: positive control. Results are mean ± SEM, *n* = 3, **p < 0.01.

Further analysis demonstrated that PS-COOH did not affect the THP-1 cell proliferation, whereas PS-NH_2_ particles virtually immediately terminated the cell division [41]. It is also notable, that the cell size decreased after treatment with positively charged PS-NH_2_ particles (Figure 3). We have previously shown that neither PS-COOH nor PS-NH_2_ polystyrene nanoparticles affect cell viability when added to cells only for one day [43]. In addition, PS-COOH particles exhibit no toxic effect on macrophages, THP-1 or differentiated THP-1 after longer incubation time (Figure 4 and [41]). Similarly, other authors did observe no cytotoxicity of PS-COOH in ovarian cancer and endothelial cells [48–49]. PS-NH_2_ particles not only inhibited the proliferation of THP-1 cells but after three days of induced exposure of phosphatidyl serine on the outer membrane leaflet of THP-1 cells consistent with the induction of apoptosis (Figure 4). Camptothecin, an inhibitor of topoisomerase I, was used as a positive control. Likewise, in leukemia cells xenografted onto the chick chorioallantoic membrane in vivo, intravenous administration of PS-NH_2_ led to characteristic DNA fragmentation, which is a definite sign of apoptosis. In contrast, xenografts grown on the CAM, which were treated with PS-COOH or saline, did not show DNA fragmentation [41]. THP-1 leukemia cells were more sensitive to PS-NH_2_ compared to human macrophages, which did not show signs of apoptosis at this time point [41].

These data are in agreement with the previously proposed ‘‘proton-sponge’’ hypothesis, which refers to a continuous activation of the lysosomal proton pump, lysosomal swelling and rupture by particles carrying amino groups on their surface [20,41,50–51]. Similarly, amino-terminated dendrimers have been shown to induce holes in biological membranes [52–53]. Interestingly, the degree of toxicity of such particles is proportional to the amount of amino groups on the particle surface [54] and is inversely dependent on the particle size: smaller particles are more toxic. This phenomenon can be explained by a higher surface to volume ratio of small nanoparticles and a larger surface being in contact with biological structures. Similar results were reported for different cell types and different models, either in vitro or in vivo [20,55–59].

The increased production of ROS is often considered the most prominent cause of potential toxicity of such particles. Indeed, lysosomal destabilization is known to take part in the internal as well as the external apoptotic pathway [60]. Lysosomes contain enzymes capable of activating phospholipases, which can further damage membranes including the outer membrane of the mitochondrion. This would lead to an increased and uncontrolled ROS production, the release of cytochrome c, the activation of the caspase cascade, and apoptotic cell death. Several studies report that extracts of purified lysosomes are capable of activating directly procaspases in vitro [61–62]. Another in vitro study showed that lysosomal enzymes can truncate Bid and activate Bax, the proapoptotic members of the Bcl2 family, which regulate the permeability of the mitochondrial membrane [63].

**Figure 5:**
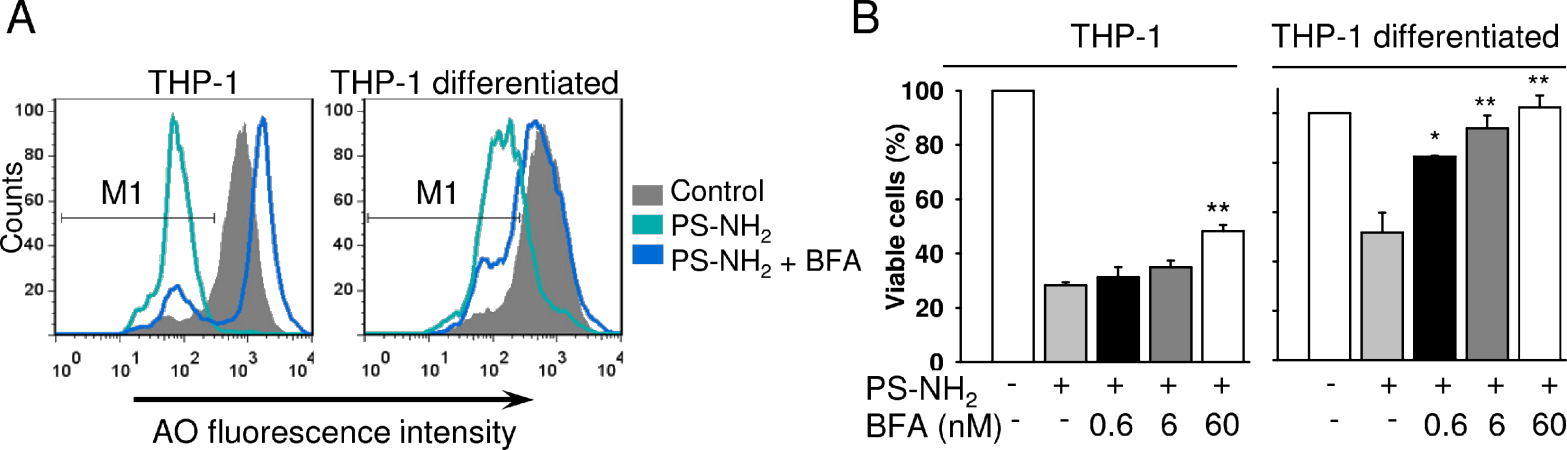
Inhibition of vacuolar ATPase by bafilomycin A1 antagonizes the toxic effect of PS-NH_2_ nanoparticles. (A) Analysis of lysosomal permeabilization of cells stimulated with PS-NH_2_ (100 µg/mL) with or without bafilomycin A1 (BFA, 6 nM) for 72 h. After treatment with nanoparticles, cells were stained with acridine orange (AO) and analyzed by flow cytometry. M1 gating was used to assess the number of AO^low^ cells with leaky lysosomes. (B) Analysis of cell viability. Cells were treated as in (A) and analyzed by XTT assay. Results are mean ± SEM, *n* = 3, *p < 0.05, **p < 0.01.

Lysosomal damage and activation of vacuolar ATPase is, indeed, central to PS-NH_2_-induced toxicity. Thus, bafilomycin A1, an inhibitor of vacuolar ATPase known to prevent acidification of lysosomal compartments [64], inhibited in a concentration-dependent manner the lysosomal rupture and apoptosis induced by a long-term exposure of THP-1 and differentiated THP-1 cells to PS-NH_2_ (Figure 5). This indicates the causative role of the lysosomal dysfunction in the apoptosis induced by nanoparticles functionalized with amino groups.

This study revealed another interesting aspect concerning the activation of the mammalian target of rapamycin (mTOR), a key kinase controlling cell growth and proliferation and implicated in many human diseases including cancer and diabetes [65]. Thus, the integrity of membranes of acidic lysosomal compartments are important for the activation of mTOR [65]. We could show that PS-NH_2_ inhibits, whereas PS-COOH activates the mTOR signaling in leukemia cells. Consistently, PS-NH_2_ inhibits the activation of the mTOR downstream targets, Akt and p70 ribosomal S6 kinase 1, and blocked proliferation in three leukemia cell lines in vitro and in vivo [41].

## Conclusion

In these studies, we have used functionalized polystyrene nanoparticles to investigate the fate of nanoparticles after their uptake by cells. Because polystyrene does not degrade in the cellular environment and shows no toxicity to cells, not even in long-term studies, the influence of the material of the nanoparticles could be largely neglected in our experiments. This feature of the nanoparticles is very important, because many studies have been performed with nanoparticles manufactured from toxic materials [21]. These studies revealed the inherent toxicity of the material of the particles, which is difficult to untangle from the effects attributed solely to size or surface properties of nanoparticles. By using polystyrene particles, it is possible to explore the effect of the size, the surface charge, and the hydrophobicity of the particles on cells. These studies show that, although, polystyrene has been claimed to be nontoxic, functionalized nanosized polystyrene particles may behave totally different from the bulk material. The surface chemistry plays a crucial role determining the impact of nanoparticles on diverse biological systems. The amino-functionalized particles can be seen as a model for cationic nanoparticles, and the carboxyl-functionalized, as a model for anionic particles. The toxicity of cationic nanoparticles might be controlled by a reduction of the amount of positively charged groups on the particle surface, by conjugation of the cationic groups with appropriate moieties to shield the positive charge and to decrease the nonspecific cellular interactions, or by replacement of reactive amine groups with amphiphilic head groups [51].

Nanoparticles manufactured from inert biocompatible polystyrene can be used to explore the effects of different surface properties on various biomedical parameters and enable the rational design of new drug delivery systems and predict functionalization-dependent health hazards that nanoparticles might exhibit.
